# Genetic Alterations Involved in Immune Escape Mechanisms of Circulating Tumour Cells in Colorectal Carcinogenesis

**DOI:** 10.1002/cam4.71683

**Published:** 2026-03-15

**Authors:** Sharmin Aktar, Matthew Masoudi, Dilpreet Moti, Vinod Gopalan, Farhadul Islam, Alfred King‐yin Lam

**Affiliations:** ^1^ School of Medicine and Dentistry Griffith University Gold Coast Queensland Australia; ^2^ Rural Health Research Institute Charles Sturt University Orange New South Wales Australia; ^3^ Department of Biochemistry and Molecular Biology Mawlana Bhashani Science and Technology University Tangail Bangladesh; ^4^ Department of Biochemistry and Molecular Biology University of Rajshahi Rajshahi Bangladesh; ^5^ Pathology Queensland, Gold Coast University Hospital Southport Queensland Australia

**Keywords:** circulating tumour cells, colorectal cancer, immune escape, immune surveillance, oncogenes, tumour suppressors

## Abstract

In colorectal cancer (CRC), circulating tumour cells (CTCs) employ genetic alterations to dodge the body's immune system. These alterations occur in specific “driver” genes, including *KRAS, BRAF, p53, MYC, APC* and *PTEN*. Changes in these genes can control how the tumour interacts with the immune system and influence the expression of immune checkpoint molecules such as PD‐1, PD‐L1, PD‐L2, CTLA‐4 and CD47. These molecules help suppress the immune system's response against the tumour, thus promoting tumour growth. However, the precise relationship between driver gene mutations and the expression of immune checkpoint molecules in CTCs, along with their clinical significance, remains incompletely understood. By studying these genetic changes and how they affect the behaviour of CTCs, researchers can gain critical insights into the development and progression of CRC, especially the roles of CTCs, which could improve CTCs' implications in liquid biopsy. Moreover, understanding these alterations can also highlight potential therapeutic targets. This may pave the way for more effective, targeted therapies to delay or prevent CRC progression. Therefore, investigating the genetic alterations in CTCs and their role in immune escape mechanisms is a significant area of study in CRC research.

## 1 Introduction

The carcinogenesis of colorectal carcinoma (CRC) is based on a stepwise aggregation of genetic and epigenetic changes, leading to either the activation of oncogenes or the deactivation of tumour suppressors. An accumulation of these genetic and/or epigenetic drift‐induced genomic instability drives CRC pathogenesis. For example, hereditary predisposition triggers the activation of proto‐oncogenes (e.g., *KRAS*) and the inactivation of tumour suppressor qualities, specifically *p53* and *APC*, which together drive the adenoma–adenocarcinoma sequence of CRC progression [1, 2].

Understanding the biology of CRC pathogenesis has driven noteworthy breakthroughs and has developed novel therapeutic targets, which have made strides for the clinical management of patients with CRC. For instance, the discovery and advancement of immune checkpoint inhibitors, such as anti‐PD‐L1 and anti‐CTLA‐4 antibodies, have demonstrated encouraging outcomes, both as standalone therapies and in combination, across various cancers, including CRC [3, 4]. There is evidence that deletion or activation of driver genes, both oncogenic and tumour suppressive, such as *KRAS*, *BRAF*, *p53*, *MYC*, *APC* and *PTEN*, might have a role in regulating tumour–immune system crosstalk and are capable of altering the expression of immune checkpoint molecules such as PD‐1, PD‐L1, PD‐L2, CTLA‐4 and CD47 etc. in a variety of malignancies [5, 6, 7, 8, 9, 10, 11].

Moreover, genetic variations in the regulatory regions of immune checkpoint genes have been identified as potential factors that might impact the immune checkpoint expression levels among individuals and the structure of immune checkpoints, thereby increasing the risk of developing cancer [12]. Furthermore, some inherited genetic markers, such as single‐nucleotide polymorphisms (SNPs), hold promise for stratifying patients into specific groups that may benefit from tailored immunotherapy approaches [12].

**FIGURE 1:**
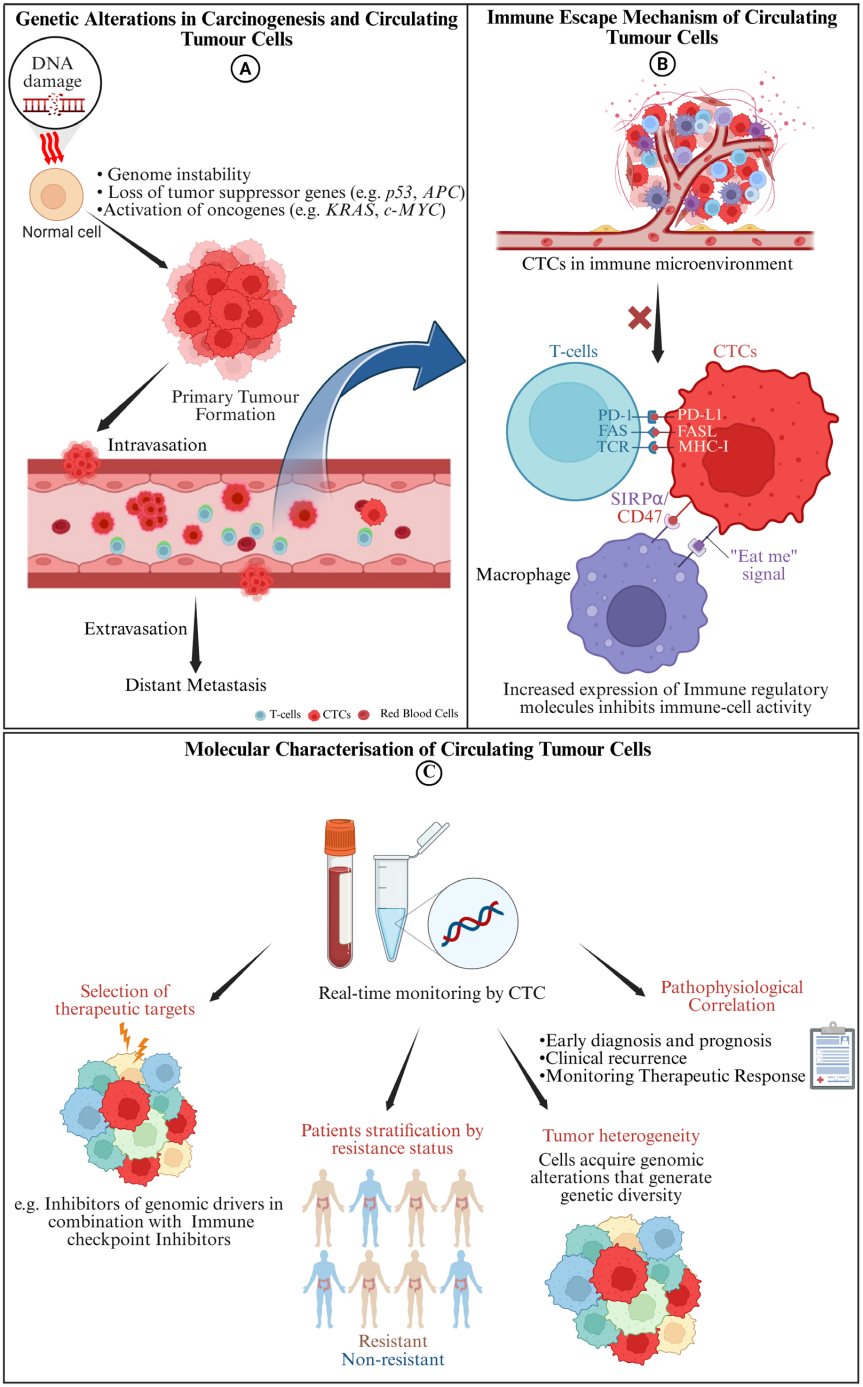
The role of circulating tumour cells (CTCs) in immune surveillance, cancer development and clinical implications of molecular characterisation of CTCs. (A) acquired gene mutations in normal cells form tumours, once released in circulation, called circulating tumour cells (CTCs), which allow them to escape from the immune surveillance system, resulting in distant metastasis. (B) Evasion of the immune surveillance system by the CTCs, for example, by increasing the expression of immune checkpoint molecules. (C) highlights the clinical significance of molecular characterisation of CTCs in immune escape mechanisms driven by cancer gene‐mediated carcinogenesis. Image created with https://BioRender.com.

An exciting development in cancer monitoring is testing for circulating tumour cells (CTCs) in blood. This minimally invasive alternative to tissue biopsy addresses its inherent limitations, such as invasiveness and late detection [13]. CTCs play a pivotal role in the metastatic cascade, partly due to their unique hereditary profiles that enhance their ability to evade immune surveillance (Figure 1) [13].

The involvement of tumour suppressor genes and proto‐oncogenes in the immune evasion strategies of CTCs through immune checkpoint molecules remains incompletely understood in the context of CRC. This review will begin by exploring how genetic alterations in tumour suppressor genes, proto‐oncogenes and immune checkpoint molecules contribute to CRC progression by facilitating immune escape. It will then examine how these mutations influence gene expression in CTCs, shedding light on the molecular heterogeneity between CTCs and primary tumours. Additionally, the review will delve into the relationship between these genetic changes and CTC behaviour, emphasising the clinical importance of genomic profiling of CTCs in CRC. A deeper understanding of these mechanisms may ultimately inform the development of targeted therapies aimed at delaying or preventing distant metastasis in CRC, and potentially other malignancies.

## 2 Overview of Common Genetic Alterations in CRC Pathogenesis

CRC is a multifactorial disease driven by a series of genetic alterations that disrupt normal cellular processes and promote uncontrolled growth and proliferation of colonic or rectal cells [14]. Hereditary modifications in many tumour suppressor genes or oncogenes are implicated in the initiation and metastasis of CRC [15]. For instance, mutations in the *Adenomatous Polyposis Coli* (*APC*) tumour suppressor gene are responsible for approximately 30%–70% of colorectal adenomas and 34%–72% of sporadic CRCs [14, 15, 16, 17]. Additionally, mutations in the *p53* gene are present in approximately 40%–50% of CRC cases [18], while alterations in the *Kirsten rat sarcoma virus* (*KRAS*) gene occur in about 40%–65% of sporadic CRCs [19, 20, 21, 22]. Furthermore, about 4% of CRC tissues with low microsatellite instability (MSI) and 40% of MSI‐high CRC tissues exhibit *BRAF* mutations [23]. Other genes involved in the TGF‐β/SMAD signalling pathway, such as *SMAD2*, *SMAD4* and *TGFRII*, are also susceptible to mutations [24, 25].

Genetic alterations in the adenoma‐carcinoma sequence involve the early inactivation of the tumour suppressor gene *APC*, followed by the later inactivation of *p53*. In contrast, mutational activation of oncogenes such as *KRAS* occurs at the intermediate stage in the process [1, 2].

**FIGURE 2:**
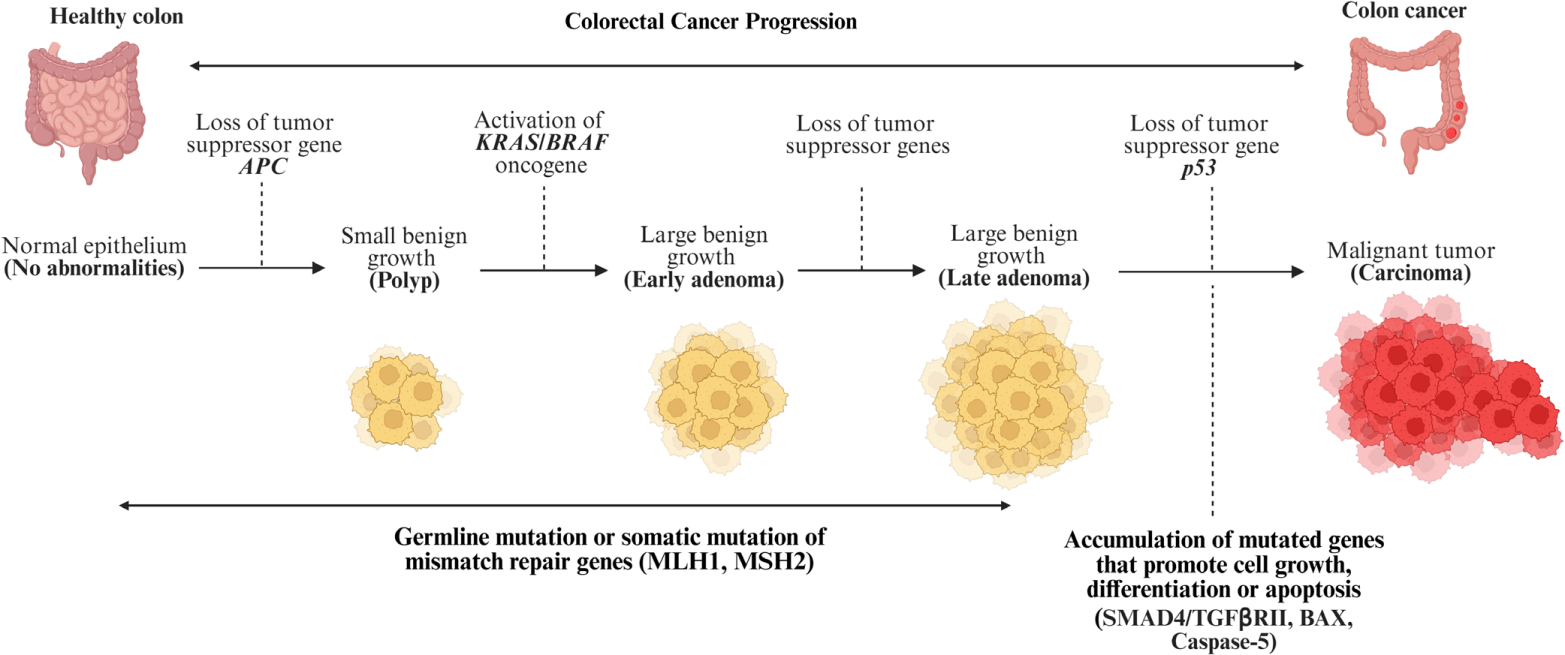
Major pathway of adenoma‐carcinoma sequence in carcinogenesis of colorectal carcinoma (CRC). Inactivation of the tumour suppressor gene APC occurs early, whereas p53 occurs late in the adenoma‐carcinoma progression sequence. In contrast, mutational activation of oncogenes such as KRAS occurs at the intermediate stage in the process [1, 2]. The concept was adopted from Kumar et al. (2020): Robbins and Cotran Pathologic Basis of Diseases. 10th Edition, Elsevier, Amsterdam. Image created in https://BioRender.com.

As previously mentioned, proper functioning of the APC is essential, as its disruption can initiate the development of CRC (Figure 2) [1, 2]. The APC protein plays a vital role in maintaining epithelial homeostasis by regulating and promoting the degradation of cytoplasmic β‐catenin [26]. APC and β‐catenin are components of the Wnt signalling pathway, a signal transduction pathway essential for colorectal tumorigenesis [26]. Notably, over 90% of CRCs have mutations that activate the Wnt pathway, with over 80% containing mutations in APC, a Wnt antagonist [27]. When *APC* is mutated, cytoplasmic β‐catenin accumulates and binds to the T‐cell factor (Tcf) family of transcription factors, altering the expression of various genes affecting proliferation, differentiation, migration and apoptosis [28]. APC regularly binds to β‐catenin, forming a complex with axin, casein‐kinase 1 (CK‐1) and glycogen synthase‐3β (GSK‐3β). This complex is then ubiquitinated and degraded intracellularly when the WNT ligand is absent/not stimulated by Wnt [28].

In cases where *APC* is mutated (as observed in many CRC cases), the protein complex loses its ability to bind beta‐catenin and direct it for degradation [29]. Thus, beta‐catenin can freely move into the nucleus and induce uncontrolled transcription of the target genes including genes involved in proliferation (*MYC, CCND1, PPARD*); stem cell fate (ASCL2); cell survival (ABCB1, BIRC5); cell differentiation (ID2, ITF2, ENC1); angiogenesis (VEGF) and cellular migration (MMP7, MMP14) [29].

The tumour suppressor p53 is well known for its role in regulating the cell cycle and, consequently, cancer progression, through its ability to induce either temporary or permanent growth arrest at the cellular level [30]. In medical literature, it is well‐established that the malignant transformation of p53 has the propensity to advance a variety of oncological diseases [31]. Mutations that inactivate p53 jeopardise cellular and chromosomal mechanisms that safeguard against aberrant and uncontrolled growth, such as cell cycle arrest, apoptosis, senescence, autophagy and cellular metabolism. Furthermore, alteration of p53 at 17p has been known to instigate the transformation of high‐grade adenoma towards adenocarcinoma (Figure 2) [1, 2, 32].

Changes in proto‐oncogenes can invigorate cancer development by driving uncontrolled cell proliferation. For instance, RAS proteins, part of the small GTPase family, act as molecular switches that regulate cellular transduction pathways, thereby playing a key role in oncogenesis. Proteins like KRAS, HRAS and NRAS play a pivotal role in the development of CRC and are commonly found to be mutated in CRC cases [33]. Over the past three decades, *KRAS* gene mutations have been recognised as a critical factor in the pathogenesis of various malignancies and are of particular importance in predicting the response to targeted therapies for CRC [34]. KRAS has been considered a possible downstream effector of the epidermal growth factor receptor (EGFR). It interacts with BRAF, triggering the activation of the MAPK (mitogen‐activated protein kinase) pathway. The abnormal activation of the MAPK pathway manifests in increased cellular proliferation and hampered apoptotic processes [24]. Furthermore, *KRAS* mutations lead to the upregulation of cyclin D1 expression, a downstream target gene, thereby further driving cellular growth and proliferation [24].

### 2.1 Alternative Genomic‐Instability Pathways Relevant to Immune Response

Beyond chromosomal instability and the canonical APC→KRAS→TP53 sequence, additional genomic instability mechanisms profoundly influence colorectal cancer (CRC) subtyping, tumour immunogenicity and response to immunotherapy. Among these, the microsatellite instability (MSI)/mismatch repair (MMR) deficiency and the POLE/POLD1 proofreading‐defect pathways are now recognised as pivotal contributors to both tumour biology and immune responsiveness [35].

Mismatch repair deficiency (dMMR) and microsatellite instability‐high (MSI‐H) arise from pathogenic alterations in key MMR genes—MLH1, MSH2, MSH6 and PMS2—or epigenetic silencing through MLH1 promoter hypermethylation. Loss of MMR function results in the accumulation of insertion–deletion mutations within microsatellite regions, producing a hypermutated phenotype characterised by high tumour mutational burden (TMB) and abundant neoantigen formation [36]. This heightened antigenicity enhances effector T‐cell infiltration and primes an interferon‐γ–driven immune microenvironment. Consequently, MSI‐H/dMMR CRCs exhibit pronounced expression of immune checkpoint molecules, including PD‐1, PD‐L1, CTLA‐4 and LAG‐3, reflecting adaptive resistance to immune activation [35, 36, 37].

Clinically, this immunogenic landscape translates into remarkable responsiveness to immune checkpoint inhibitors (ICIs). Landmark trials, such as KEYNOTE‐177, established pembrolizumab as a first‐line standard of care for metastatic MSI‐H/dMMR CRC, demonstrating superior progression‐free survival compared with chemotherapy [37]. Long‐term follow‐up and real‐world evidence have reinforced the durable benefit of anti–PD‐1 monotherapy in this subset [38, 39]. However, resistance can emerge through secondary mutations in antigen‐presentation machinery (e.g., B2M, JAK1/2) or upregulation of alternative inhibitory receptors, underscoring the complexity of immune escape even in hypermutated tumours.

A related but distinct pathway involves proofreading deficiency in the exonuclease domains of DNA polymerases epsilon (POLE) and delta (POLD1). Tumours harbouring POLE/POLD1 mutations exhibit an “ultramutated” genotype with exceedingly high TMB (> 100 mutations/Mb) and distinctive C>A and T>G mutational signatures [35]. These alterations induce strong cytotoxic lymphocyte infiltration and upregulation of immune effector genes, yet the rarity of such mutations (< 2% of CRCs) limits their clinical characterisation. Pooled analyses of retrospective and prospective cohorts suggest that POLE/POLD1‐mutant CRCs may achieve even higher and more durable responses to ICIs compared with dMMR/MSI‐H tumours [35].

Mechanistically, these hypermutated pathways converge on genome‐instability‐induced immunogenicity, linking defective DNA repair or proofreading to immune checkpoint upregulation and enhanced tumour–immune interplay. Notably, such mechanisms may influence the phenotypic and molecular profiles of circulating tumour cells (CTCs). CTCs derived from dMMR or POLE‐deficient tumours could retain hypermutation‐associated neoantigen expression and display heightened checkpoint molecule expression, potentially serving as liquid‐biopsy biomarkers for ICI sensitivity. Integration of MSI/MMR and POLE/POLD1 status into CTC analyses could therefore refine patient stratification and therapeutic monitoring.

Collectively, these alternative pathways underscore the intersection between DNA‐repair fidelity, mutational landscape and immune dynamics in CRC. Their incorporation into CTC‐based research and immunogenomic profiling holds promise for advancing precision immuno‐oncology. As Masoudi et al. (2024) summarised, metabolic and genomic plasticity are central to tumour adaptation under immune and therapeutic pressure, a principle that equally applies to the immune evolution of hypermutated CRC clones in circulation [40].

## 3 Evasion of Tumour Immune Surveillance in CRC Carcinogenesis: Insights Into Immune Checkpoint Molecules

Tumour cells exploit immune checkpoint pathways to evade immune surveillance, thereby suppressing antitumor immune responses [41]. Among them, immune checkpoint ligands (and their associated receptors), such as PD‐L1/PD‐1, galectin‐9/TIM‐3, IDO1, LAG‐3 and CTLA‐4, play a foundational role in tumour‐induced immunosuppression [38]. Dysregulation of these checkpoint molecules dually compromises the integrity of tumour suppression mechanisms, further promoting CRC carcinogenesis. In recent research and development of cancer treatments, checkpoint molecules have become a highlighted focus of potential future immunotherapies, given the temporal sustainability of their effects [39, 42, 43, 44].

Programmed cell death‐1 (PD‐1, also known as CD279) is a key inhibitor of the cytotoxic immune response and is expressed on CD4+ T cells, CD8+ T‐cells, NKT cells, B‐cells and monocytes/macrophages [45]. One of its ligands, PD‐L1 (CD274 or B7‐H1), is overexpressed on the surface of various tumour cells, undermining immune defence and promoting immune resistance [45, 46]. The expression of PD‐L1 can be initiated by an assortment of cytokines and exosomes within the tumour microenvironment, which increases the PD‐L1/PD‐1 flag to diminish the cytotoxicity of T‐cells and, hence, advance tumour evasion [38, 45]. PD‐1 was astoundingly upregulated on CD8+ T‐cells within the tumour microenvironment of CRC cases compared to CD8+ tumour‐free lymph nodes [39]. Additionally, the level of PD‐L1 expression on CRC appeared to be pivotal within the inhibition of cytokine generation within the tumour microenvironment [45]. Notably, studies have shown that cancer cells from solid tumours can upregulate the expression of PD‐1 ligands, delivering inhibitory signals that downregulate T‐cell activation, ultimately suppressing immune responses [47] and inducing immune tolerance [48]. Expression of PD‐1 ligands on tumour cells was also shown to suppress the cytolytic activity of CD8^+^ T‐cells [49, 50]. Indeed, PD‐L1 is expressed in different tumours [51, 52, 53]. Most importantly, a significant correlation has been observed between the expression of PD‐1 ligands on tumour cells and a poor prognosis [50].

Cytotoxic T lymphocyte antigen‐4 (CTLA‐4) has been recognised as another factor in CRC development due to its ability to restrain the T‐cell response. Initial studies revealed that CTLA‐4 is expressed in tumour‐infiltrating lymphocytes, leading to the suppression of anti‐tumoral immune responses. However, recent findings have demonstrated that CTLA‐4 can also be expressed on tumour cells and shield tumour cells from immune responses [54]. Indeed, the CTLA‐4 expression has been shown in other cancers, such as non‐small cell lung cancer, melanoma and CRC, functioning similarly as a means of immunological tolerance throughout cancer progression [55]. In a recent study, CTLA‐4 expression was assessed in CRC tissues and colon cancer cell lines (HT‐29, HCT‐166 and SW480). According to the study's findings, the overexpression of CTLA‐4 is particularly pronounced in CRC tissues relative to adjacent non‐neoplastic tissues [56]. In vitro studies have demonstrated that the chemotherapeutic drug (capecitabine) can significantly reduce the gene expression of CTLA‐4 in colon cancer cells, SW480 cells, potentially linking chemotherapy with immunotherapy in CRC. Blocking this inhibitory molecule can re‐activate immune cells, particularly T cells, in CRC patients, enhancing their ability to combat the tumour [56].

Liu et al. (2017) illustrate CD47, yet another checkpoint molecule of interest. CD47, a ubiquitous cell surface glycoprotein, is among a superfamily of immunoglobulins [57]. CD47 expression has been shown to promote immunological evasion of tumour cells [58]. It is demonstrated that T‐cells are essential for CD47‐mediated tumour regression. CD47 could escape phagocytosis upon binding to its ligand signal‐regulatory protein α (SIRPα), expressed on macrophages and dendritic cells (DCs) [59]. A pronounced upregulation of the *CD47* gene in CTCs from patients with CRC, as compared to corresponding primary tumour tissues, was found to play a potential role in the immune escape mechanism, which may be responsible for the survival of CTCs in circulation [60]. The *CD47* gene may not be expressed in the primary tumours but can be overexpressed during the development of bone metastasis, indicating that CD47 expression could play a role in the initiation of metastatic spread [58].

Other immune checkpoint molecules, such as PD‐L2, B7‐H3, IDO1 (Indoleamine 2,3‐dioxygenase), TIM‐3 (T cell immunoglobulin and mucin domain‐containing protein 3) may also regulate immune‐related pathways and has been studied in different cancers [38, 52, 61]. For example, Zhao et al. (2019) identified immune‐related biomarkers (CTLA‐4, PD‐L1 and PD‐L2) and potential therapeutic targets to enhance understanding of the immunological landscape in prostate cancer [62]. Zhao et al. (2019) also showed significant correlations with immune‐related pathways on gene set enrichment analysis. Wang et al. demonstrated that PD‐L2 is expressed in approximately 40% of CRCs, and its expression is independently associated with poor survival of CRC patients [63]. In a study among 124 CRC cases, Wang et al. utilised immunofluorescence to reveal a significant association between PD‐L2 overexpression in cancer cells and worse overall survival (46.3 months versus 69.1 months; *p* = 0.0004). They also revealed that PD‐L2 overexpression in CRC cells, under the regulation by IFNγ and glycosylation, is associated with poor survival of patients with CRC. These findings highlight PD‐L2 as a promising therapeutic target in CRC and suggest potential routes to control PD‐L2 expression in CRC cells [63].

## 4 Circulating Tumour Cells (CTCs)

### 4.1 Background, Cytomorphology and Biology

Circulating tumour cells (CTCs) are a population of cells first identified and proposed by Thomas Ashworth as the seeds of tumour cell dissemination and metastasis [64]. Specifically, ‘CTCs’ refer to single cells that enter and circulate in the bloodstream, whereas a cluster of circulating CTCs is referred to as circulating tumour micro‐emboli (CTMs); disseminated tumour cells (DTCs) is the term used for such cells in the bone marrow [65]. CTCs are rare, with only one CTC present among 10^6^–10^7^ leukocytes. They circulate through body fluids and spread to various organs, serving as seeds for secondary tumours [66]. The precise morphological characteristics of CTCs have not yet been elucidated; however, it is agreed that CTCs have a relatively larger, irregular and dense basophilic nucleus than white blood cells and express specific surface markers [67].

Epithelial‐mesenchymal transition (EMT) is a normal physiological phenomenon that naturally occurs during embryogenesis. During EMT, epithelial cells lose their ability of cell‐to‐cell adhesion through the loss of epithelial and cell adhesion markers. Additionally, they also lose their apical‐basal polarity and obtain mesenchymal markers. In a concerted manner, these changes allow the cells to migrate and intravasate through the extracellular matrix (ECM) membrane into the circulation as CTCs. Within the blood vessel, CTCs regain their epithelial phenotype through a reverse process of EMT known as mesenchymal‐epithelial transition (MET), which permits the cell to seed and metastasise at a secondary site. Thus, tumour cells disseminate from their epithelial origin to colonise distant organs via the EMT and MET processes [68].

### 4.2 Immune Checkpoint Molecules Support CTCs to Escape From Immune Surveillance

The prognosis of an individual's diagnosis of CRC is closely determined by the occurrence of distant metastases [60]. To form metastasis, CTCs must detach from the primary tumour, survive in the bloodstream and colonise distant secondary sites [69]. Various factors either promote or hinder the entry and survival of CTCs in the bloodstream, as well as the activation of dormant disseminated tumour cells [59]. Among them, immune checkpoint molecules (PD‐1, PD‐L1, PD‐L2, B7‐H3, CTLA‐4 and CD47) have been reported to contribute to the ability of CTCs to evade immune surveillance [58, 67].

CTCs have the potential to exploit a variety of new pathways to avoid immune surveillance through mechanisms that alter the expression of MHC molecules, NK‐cell ligands and FAS/FAS ligand (FASL) [59]. CTCs can mediate the downregulation or complete loss of MHC I expression to evade death by the cytolytic action of T lymphocytes [70]. NK cells mediate their cytotoxic activity via NK cell receptor D (NKG2D) interacting with MHC I polypeptide‐related sequence A or B (MICA/MICB), which are expressed on neoplastic cells [71]. Downregulation of MICA/MICB in stem‐like breast cancer is mediated by aberrant expression of an oncogenic microRNA, miR20a [72]. CTCs thus can interfere with the cytotoxic activity of immune cells by inducing downregulation of the NKG2D expression or MHC class I expression, which can lead to increased expression of immune checkpoint molecules.

PD‐1 and its ligand PD‐L1, the inhibitory immune checkpoint molecules, target tumour‐specific effector T cell‐induced immunosuppressive pathways [73]. PD‐L1 is expressed by tumour cells in the tumour microenvironment and transmits inhibitory signals via PD‐1 expressed on T cells, thereby limiting immune effector functions. Through this mechanism, CTCs could evade the immune response, facilitating metastasis. CTLA‐4A, another immune checkpoint molecule typically expressed in T cells, could demonstrate antitumour immune responses. In a pioneering study, a group of researchers characterised the expression of CTLA‐4 among other immune checkpoint molecules on CTCs in men with prostate cancer [61]. The specific mechanism through which CTLA‐4 aids CTCs in evading immune surveillance remains unknown and warrants further investigation. CTCs also evade the host immune system by expressing CD47, which signals for the inhibition of phagocytosis. The upregulation of CD47 was considered a potential immune‐escape mechanism, enabling the CTCs to exist in a dormant state [60].

### 4.3 Tumour Microenvironment (TME) Factors Licencing CTC Immune Evasion

The emergence and persistence of circulating tumour cells (CTCs) are intimately shaped by the tumour microenvironment (TME), a dynamic ecosystem comprising stromal elements, immune cell infiltrates and soluble mediators that collectively dictate the immune landscape of colorectal cancer. Within this niche, a continuous exchange of signals between malignant and non‐malignant components confers selective pressures that promote the acquisition of immune‐evasive traits [58].

Immunosuppressive cellular populations such as regulatory T cells (Tregs) and myeloid‐derived suppressor cells (MDSCs) actively blunt cytotoxic T‐lymphocyte and natural killer (NK)‐cell function through secretion of IL‐10, TGF‐β and reactive oxygen species, as well as expression of checkpoint ligands. Tumour‐associated macrophages further potentiate these effects by delivering CD47–SIRPα “don't‐eat‐me” signals that impair phagocytic clearance, thereby facilitating tumour‐cell intravasation and survival in the circulation. Sustained antigen exposure within the TME fosters a state of chronic T‐cell activation and exhaustion, typified by co‐expression of inhibitory receptors such as PD‐1, TIM‐3, LAG‐3 and TIGIT—hallmarks of ineffective antitumour immunity frequently mirrored in the immune signatures of CTCs.

Cytokine and chemokine signalling networks reinforce these processes. Elevated levels of TGF‐β, IL‐6 and IL‐10 promote epithelial–mesenchymal transition (EMT), suppress effector T‐cell recruitment and drive enrichment of CTC populations with stem‐like and migratory phenotypes. In parallel, activation of the CXCL12–CXCR4 axis enhances motility and vascular adhesion, supporting intravasation and systemic dissemination. These signalling cascades converge to generate a permissive immunological milieu from which immune‐refractory CTCs emerge [59].

A critical additional layer of regulation arises from metabolic and hypoxic adaptations within the TME. Hypoxia‐inducible factor 1α (HIF‐1α) upregulates PD‐L1 expression and orchestrates metabolic reprogramming that favours glycolytic flux over oxidative phosphorylation [60]. This metabolic shift not only provides bioenergetic flexibility under nutrient‐limited conditions but also suppresses antitumour immune responses through lactate accumulation and acidification of the microenvironment. As demonstrated by Masoudi et al. (2024), metabolic plasticity of cancer stem cells (CSCs) allows dynamic oscillation between glycolytic and oxidative states, enabling survival under immune and therapeutic stress while sustaining invasive potential. These CSC‐like populations, frequently represented among CTCs, thus serve as key mediators of immune evasion and metastatic competency in CRC [40].

Understanding the reciprocal interplay between TME composition, cytokine and metabolic cues and checkpoint expression on CTCs is essential for accurate interpretation of immune signatures in liquid‐biopsy assays. Integrating TME‐derived biomarkers—such as immune‐cell ratios, hypoxia indicators and cytokine profiles with CTC phenotyping may substantially enhance prognostic precision and improve prediction of immunotherapy responsiveness in colorectal cancer.

## 5 Genetic Variations in Immune Checkpoint Molecules

Genetic polymorphisms, particularly single‐nucleotide polymorphisms (SNPs) in immune checkpoint molecules, have been explored in the context of various cancers and shown to be associated with cancer risk through genome‐wide studies [12]. SNPs have the potential to alter protein structure and modify gene transcription through the interplay of transcription factors, histone‐binding and DNA looping [74]. As blueprints of the cell, an understanding of the factors that influence gene expression, particularly those that act as immune checkpoint molecules, can be insightful in the effort to overcome cancerous pathology.

Over the past few decades, the discovery of immune checkpoint molecule SNPs has led to significant efforts to explore the connection between a host's genetic background and different types of cancer [75, 76, 77, 78, 79, 80, 81]. The involvement of the immune checkpoint inhibition pathway in CRC pathogenesis was highlighted by studies showing a correlation between single nucleotide polymorphisms in the immune checkpoint molecules in the context of CRC in both a Chinese [75] and Iranian population [78], suggesting that genetic variation in co‐inhibitory molecules may be associated with cancer risk. Ge et al. hypothesised that these polymorphisms might activate new splice sites in probable splice sites and splicing enhancer motifs while destroying a splice site in silencer motifs [75]. Consequently, these variations may boost the splicing signal and CTLA‐4 and BTLA (*B‐ and T‐lymphocyte attenuator*) expression. Sun et al. showed that a slight alteration in CTLA‐4 activity, caused by an SNP leading to an amino acid substitution, could impair T‐cell proliferation by reducing co‐stimulation signalling [79].

Polymorphisms in *PD‐1* and *PD‐L1*, like those in *CTLA‐4*, can lead to increased expression, reducing T‐cell activation and proliferation, suggesting that these alleles may be risk factors for cancer [82, 83]. Polymorphisms in *CD47* were also found to be associated with distant metastasis and CRC survival [76].

### 5.1 Epigenetic Mechanisms That Facilitate Immune Evasion

Epigenetic alterations—DNA promoter hypermethylation, histone modifications and non‐coding RNAs (miRNAs/lncRNAs)—reshape tumour–immune interactions in CRC [74]. Examples include: (i) MLH1 promoter hypermethylation that drives dMMR/MSI‐H; (ii) methylation‐mediated silencing of antigen‐presentation genes (e.g., HLA class I components), impairing CD8^+^ T‐cell recognition; (iii) chromatin‐state changes and miRNA networks (e.g., miR‐based regulation) that modulate PD‐L1 transcription/stability; and (iv) hypoxia‐responsive epigenetic programmes converging on checkpoint expression [40]. Early trials and preclinical work suggest DNA methyltransferase (DNMT) and histone deacetylase (HDAC) inhibitors can restore antigen presentation and enhance PD‐(L)1 efficacy, motivating epigenetic–immunotherapy combinations in CRC [75].

## 6 Interplay of Carcinogenesis‐Associated Genes in Orchestrating the Antitumour Immune Response

A dynamic interaction exists between factors that promote cancer development and the body's immune response against tumour formation. Genetic alterations in tumour cells can generate an immunosuppressive microenvironment, thereby hindering the immune system's ability to target and address pathogenetic processes. A key aspect of this phenomenon is the role of immune checkpoint molecules.

Table 1 summarises the relevant research exploring the role of genetic alterations in key tumour suppressor genes and oncogenes in immune checkpoint‐mediated immune evasion across various cancers. The following section will highlight how genetic alterations in tumour cells affect the pathways of immune checkpoint inhibition.

**TABLE 1:** Studies demonstrate the role of tumour suppressor genes, oncogenes and immune checkpoint molecules in the immune escape mechanism in colon cancer.

Study	Cancer type	Driver gene mutation	Immune checkpoint molecules	Key findings
Guo et al. 2017 [84]	NSCLC	*KRAS*	PD‐L1	*KRAS* mutations influence PD‐L1 expression in NSCLC via activation of the PI3K/AKT and MEK/ERK pathways, suggesting a link between driver gene mutation and tumour immune evasion.
Lastwika et al. 2016 [85]	NSCLC	*EGFR*, *KRAS* and *ALK*	PD‐L1	By increasing PD‐L1 expression, oncogenic activation of the AKT–mTOR pathway aids in immune evasion.
Chen et al. 2017 [86]	NSCLC	*KRAS*	PD‐L1	1. KRAS‐mediated up‐regulation of PD‐L1 through p‐ERK signalling. 2. Induced the apoptosis of CD3‐positive T cells.
Coelho et al. 2017 [87]	Human epithelial cell line	*KRAS*, *NRAS*	PD‐L1	Tumour cells might upregulate PD‐L1 expression in response to activation of the RAS pathway and evade immune attack.
Liu et al. 2021 [88]	CRC	*KRAS*	PD‐L1, CTLA4 and TIM‐3	*KRAS* mutations were found to drastically downregulate immune checkpoint‐associated molecules, suggesting that these mutations may inhibit checkpoint molecules.
Fu et al. 2020 [7]	Colon cancer	*KRAS*	BTLA, CD80, CD86, CTLA4, IDO1, PD‐L2 and TIGIT	BTLA, CD80, CD86, CTLA4, IDO1, PDCD1LG2 and TIGIT showed reduced expression in *KRAS* mutation carriers.
Glorieux et al. 2021 [6]	Pancreatic cancer	*KRAS*	PD‐L1	*KRAS* promotes PD‐L1 expression and suggests that modulation of ROS or inhibition of the FGFR1 pathway could be a novel strategy to abrogate PD‐L1‐mediated immunosuppression.
Casey et al. 2016 [89]	—	*MYC*	CD47 and PD‐L1	MYC inactivation in mouse tumours down‐regulated CD47 and PD‐L1 expression and enhanced the antitumour immune response, while MYC inactivation with enforced expression of CD47 or PD‐L1 suppressed the immune response.
Zou et al. 2018 [9]	HCC	*MYC*	PD‐L1	The inhibition of MYC elevated the expression of STAT1, a critical component of the IFN‐γ signalling pathway, leading to the elevation of PD‐L1 expression in HCC cells exposed to IFN‐γ.
Kim et al. 2017 [90]	NSCLC	*MYC*	PD‐L1	MYC expression significantly correlated with PD‐L1 expression.
Cortez et al. 2015 [91]	NSCLC	*p53*	PDL1	p53 regulates PDL1 via miR‐34, reducing the number of radiation‐induced macrophages and T‐regulatory cells.
Albitar et al. 2017 [92]	CRC	K‐RAS or *p53*	PD‐L1	PD‐L1 expression is significantly more common in CRC, lacking mutations in *RAS* or *P53*.
Thiem et al. 2019 [5]	Melanoma	*p53*	PD‐L1	For *P53*‐mutated tumours, an increased CD274 mRNA expression and a higher frequency of PD‐L1 positivity were observed.
Cha et al. 2016 [10]	NSCLC	*p53*	PD‐L1	1. PD‐L1 expression in tumour cells was correlated with. 2. PD‐L1 expression was associated with p53 aberrant expression and showed poor prognosis.
Biton et al. 2018 [93]	NSCLC	*p53, STK11* and *EGFR*	PD‐L1	The presence of *P53* mutations without co‐occurring *STK11* or *EGFR* alterations, independently of *KRAS* mutations, identified the group of tumours with the highest CD8 T‐cell density and PD‐L1 expression.
Cen et al. 2021 [94]	Colonic epithelial cell lines, mouse colon tissue	*APC*	PD‐L1	Mutations in the *APC* gene lead to colonic epithelial cell resistance to CD8+ T cell cytotoxicity by induction of PD‐L1 expression.
Rosenbaum et al. 2016 [95]	CRC	*BRAF*, *KRAS*	PD‐L1	PD‐L1 expression was associated with increased CD8 and tumour‐infiltrating lymphocytes, *BRAF* mutation and a lower frequency of *KRAS* mutation.
Parsa et al. 2007 [96]	Glioma	*PTEN*	PD‐L1	Expression of PD‐L1 increases after the loss of PTEN and activation of the PI3K pathway.
Song et al. 2013 [97]	CRC	*PTEN*	PD‐L1	A high level of PD‐L1 expression was associated with increased risks of metastatic progression.

Abbreviations: CRC, colorectal carcinoma; HCC, hepatocellular carcinoma; NSCLC, non‐small cell lung carcinoma.

### 6.1 p53

The genetic instability of the *p53* gene in cancer cells can modulate the tumour biology of cancer cells and the host immune response [98]. Loss of *p53* in cancer cells can enhance the tumour‐supporting activity of different immune cell subpopulations, such as myeloid cells, neutrophils, macrophages, monocytes and T‐regulatory cells (Tregs) by inhibiting the effector functions of CD4^+^ and CD8^+^ T‐cells [99]. Recent research has revealed agonistic relationships between *p53* and immune checkpoints [5, 10, 93, 100, 101]. For example, *p53* suppresses PD‐L1 function by upregulating the expression of PD‐L1 via the microRNA, miR‐34a [91]; miR‐34a, which is regulated by p53, acts as a suppressor of PD‐L1 expression. When p53 activity is lost, the production of miR‐34a is reduced or impaired, thereby downregulating major histocompatibility complex class I (MHC I) and natural killer group membrane D/ligands (NKG2D/NKG2DL). As a result, PD‐L1 expression on the surface of cancer cells increases the propensity for cancer cells to evade the host's immune response, thereby promoting tumour growth and progression.

### 6.2 APC

Mutation of *APC* may also contribute to the ability of a tumour to evade the immune response through its implications in the immune checkpoint pathway [94]. *APC* is involved in the regulation of intestinal inflammation and, thereby, the progression of adenocarcinoma; these processes are the products of impaired differentiation and the circulating cytokines (Nuclear Activated T Cell Factor (NFAT)‐regulated cytokines, particularly IL‐10) [102]. Despite this, the molecular mechanisms linking APC depletion or activation to CRC carcinogenesis and their impact on the expression of immune checkpoint molecules remain unexplored [103]. However, it can be hypothesised that APC may regulate CTLA‐4 expression, as previous studies have shown a correlation between CTLA‐4 expression and the suppressive activity of Tregs [103]. Recently, for the first time, demonstrated that *APC* mutation‐induced PD‐L1 expression in colon epithelial cells enhances their resistance to cytotoxicity by CD8+ T cells. This study broadens our understanding of APC's role in CRC by uncovering a novel consequence of APC depletion in tumour immune evasion [94].

### 6.3 KRAS

*KRAS* is one of the most mutated oncogenes in cancer, being responsible for tumorigenesis and serves as a predictive biomarker in cancer treatment. Recent studies revealed the crucial role of *KRAS* activation in mediating the crosstalk between cancer and immune cells, promoting the shift from an anti‐tumourigenic to a pro‐tumourigenic state, thereby inducing immunosurveillance evasion of cancer cells [104]. *KRAS* mutation is found to be associated with immunosuppression in CRC [88]. *RAS* mutation in CRC downregulates the IFN‐γ pathway, leading to limited CD8^+^ T cell activation [88]. *KRAS* activation can facilitate immune evasion by downregulating MHC class I antigen molecules on the cell surface, thus reducing the ability of CD8^+^ cytotoxic T‐cells to recognise and target cancer cells [104]. These events contribute to the establishment of an immunosuppressive microenvironment, partly mediated by the increased expression of T‐cell exhaustion markers, including PD‐1, CTLA‐4 and *TIM‐3*.

Another study revealed that *KRAS* mutations in lung cancer cause PD‐L1 upregulation, which is mediated through ERK signalling [86, 105]. The relationship between *KRAS* activation and the expression of PD‐L1 and PD‐1 has also been investigated in various cancers, including CRC. In the case of pancreatic cancer, the activation of *KRAS* has been demonstrated to be associated with increased expression of PD‐1 [106]. In CRC, *KRAS* mutations are associated with low expression of PD‐L1 and hence predict poor immune infiltration [92, 95, 107]. Oncogenic *KRAS* can also trigger an immunosuppressive environment in CRC by limiting interferon regulatory factor 2 (IRF2) expression, which leads to enhanced expression of CXCL3 on cells, promoting their recruitment to the tumour microenvironment, as well as restricted T cell accumulation and subsequent resistance to immune checkpoint inhibitors [108].

### 6.4 MYC

MYC may also appear to initiate and maintain tumourigenesis in part through the modulation of immune regulatory molecules [108]. A recent study showed that *c‐MYC* regulates the expression of PD‐L1, which suppresses the adaptive immune response and CD47, which inhibits the innate immune response [107]. This study suggested that exogenous overexpression of PD‐L1 and CD47 on cancer cells restricted the recruitment of CD4^+^ T cells and macrophages to the tumour. Another study explored whether *MYC* plays a role in regulating PD‐L1 expression induced by IFN‐γ in hepatocellular carcinoma (HCC) cells [9]. Interestingly, they found that the knockdown of *MYC* expression enhanced PD‐L1 expression induced by IFN‐γ in hepatocellular carcinoma cells. The downregulation of *MYC* increases the expression of STAT1, resulting in elevated activation of IFN‐γ receptor signalling [9]. These findings imply that suppression of *MYC* activity might promote immune evasion mediated by the PD‐L1 molecule in hepatocellular carcinoma [9].

### 6.5 Others

Other driver genes, such as *EGFR*, *PTEN* and *STAT3*, have been shown to regulate the expression of immune checkpoints [96, 97, 109, 110, 111, 112, 113]. It is proposed that using immune checkpoint inhibitors for EGFR and PTEN status provides a novel therapeutic strategy for cancer patients, enhancing the efficiency of clinical outcomes. For example, a study investigated the clinical significance of PD‐L1 in a subset of 39 patients with *PTEN* loss to explore whether the effect of PD‐L1 in CRC depends on *PTEN* expression [97]. They demonstrated that the PD‐L1 protein level was increased in CRC cells treated with siRNA PTEN. The ability of PTEN to regulate PD‐L1 expression was unaffected by IFN‐γ, the primary inducer of PD‐L1 production. PTEN and IFN‐γ likely use different signalling mechanisms to control PD‐L1 expression.

## 7 Liquid Biopsy: Circulating Tumour Cells Versus Circulating Tumour DNAs

Among the growing recognition of precision medicine that incorporates genetic, environmental and lifestyle considerations, liquid biopsy has emerged as a new approach to personalised treatment. A liquid biopsy is a minimally invasive diagnostic technique that analyses various biomolecules (such as CTCs, cell‐free DNA (cfDNA) and extracellular vesicles) that may be present in the blood, urine or other bodily fluids [114]. This technique has been used for the early detection, diagnosis and monitoring of cancer, as well as for evaluating treatment efficacy and detecting treatment‐resistant mutations.

Liquid biopsy has several advantages over traditional tissue biopsy, including reduced invasiveness, the ability to detect mutations that may not be present in the primary tumour or metastases, and the potential for real‐time monitoring of disease progression. CTCs and circulating tumour DNAs (ctDNAs) are the two biomarkers in liquid biopsy that have been studied the most extensively. Insights gained from studying CTCs and ctDNA significantly enhance our understanding of cancer biology and tumour evolution, as well as the effectiveness of cancer treatments and the emergence of therapy resistance. Previously published reports have reviewed the strengths and weaknesses of existing CTC and ctDNA studies thus far [115, 116].

The use of ctDNA as a marker for metastatic cancers shows promise, with emerging evidence suggesting that it is more sensitive and suitable than using CTCs as a biomarker [117, 118]. ctDNA can be released into the bloodstream during tumour cell apoptosis or necrosis, making it a suitable tool for determining disease stage, predicting residual disease and recurrence and establishing an early cancer diagnosis [117, 118]. However, technical challenges and obstacles related to the low signal‐to‐noise ratio caused by high levels of cell‐free DNAs, as well as the low frequency of certain genomic aberrations and interference from non‐neoplastic clonal expansion, may affect the accuracy of cancer detection through this approach [114, 119, 120, 121, 122].

Alternatively, CTCs have numerous advantages over other liquid biopsy techniques. Although the low detection rate of CTCs limits their current use in therapeutic settings, several high‐sensitivity isolation methods have been developed in recent years to overcome this challenge, including CellCollector and CytoSorter [119]. Recent research by Morgan et al. has shown that a cut point of 675 CTCs per 7.5 mL of peripheral blood obtained by surface‐enhanced Raman scattering (SERS) has the best combination of sensitivity and specificity for predicting the emergence of metastasis [123]. A benefit of studying CTCs is that they can be molecularly characterised to map the tumour biology of the individual subpopulations of cells that make up the CTCs, such as epithelial, mesenchymal and stem cell markers [115]. Also, CTCs can potentially build 3‐dimensional organoid cultures, which can be expanded for functional testing or drug‐screening assays [119]. Due to the extensive heterogeneity in tumour subpopulations, single‐cell analysis of CTCs helps reduce this heterogeneity, making it a powerful tool for understanding mechanisms behind drug resistance, metastasis and treatment response [124].

CRC is a complex and heterogeneous disease, and the molecular characterisation of immune regulatory molecules in CTCs can provide critical information regarding disease prognosis, treatment selection and the development of new immunotherapeutic strategies. The expression of immune checkpoint molecules, such as PD‐L1, in CTCs is associated with a poorer prognosis [125]. Moreover, Steinert et al. (2017) identified an overexpression of *CD47*, a molecule that promotes immune system evasion via CTCs in patients with CRC, suggesting that targeting CD47 may be a promising approach for enhancing the anti‐tumour immune response [60]. Molecular characterisation of CTCs thus provides valuable insights into the mechanisms of immune evasion, tumour‐host interactions and the development of resistance to immunotherapy.

The remainder of this review will focus on the biology of CTCs and the knowledge gap in the currently available literature regarding the clinical importance of molecular characterisation of immune regulatory molecules in CTCs related to CRC carcinogenesis.

## 8 Clinical Significance of Molecular Characterisation of CTCs in CRC Carcinogenesis

Due to the poor response rates and short duration of efficacy with targeted therapies in patients harbouring driver gene mutations, the development of immune checkpoint inhibitors, such as anti‐PD‐L1 and anti‐CTLA‐4 antibodies, has shown promising results in both monotherapy and combination therapy, across the frontline and subsequent treatment lines for various cancers, including CRC [86, 126]. However, the high variability in biomarker expression, driven by the inherent heterogeneity of cancer cells, diminishes the effectiveness of personalised treatments based on biomarkers assessed from the original tumour at diagnosis [127]. This has led to increasing interest in the development of non‐invasive technologies for cancer diagnosis and treatment.

CTCs offer significant potential as a minimally invasive and reproducible platform for monitoring the dynamic progression of the disease. Emerging studies have already highlighted the clinical value of CTCs in peripheral blood as diagnostic, prognostic and predictive biomarkers in multiple malignancies [128, 129, 130, 131, 132, 133, 134, 135, 136]. However, several studies have attempted to account for the fact that CTCs harbouring driver genes such as *p53* and *KRAS* mutations are dissimilar to their matched primary tumours [127, 137, 138, 139, 140]. Intratumour heterogeneity is one of the reasons for this discordance, suggesting that both wild‐type and mutant subpopulations of cells may coexist within the same tumour and compete with one another for shedding into the bloodstream. CTC heterogeneity might also contribute to resistance to targeted therapy. For example, TP53 mutations in CTCs have been shown to predict a poor response to neoadjuvant chemotherapy [141, 142, 143].

Further, CTCs from numerous malignancies, including breast, prostate, colorectal, lung, urothelial, head and neck cancers, have been shown to express immune checkpoint molecules and are associated with poor prognoses [5, 73, 144, 145, 146, 147, 148, 149]. For instance, Nicolazzo and colleagues investigated the expression of PD‐L1 in CTCs from 24 patients with metastatic non‐small cell lung cancer (NSCLC) treated with nivolumab, and they found that PD‐L1 expression serves significant predictive value several months after the onset of therapy [146]. Gene expression profiling also revealed that PD‐L1, PD‐L2, B7‐H3 and CTLA‐4 are expressed heterogeneously, suggesting that identifying these immune checkpoints may facilitate monitoring the patient's response to immunotherapy [61]. Patients with CRC had CTCs that overexpressed the *CD47* gene compared to their primary tumour tissues, which may have a prognostic value [60]. CD47 expression was also found in CD44^+^ CTCs from a patient with advanced metastatic breast cancer (BC). The patient, whose initial tumour was CD47‐negative, later developed a bone metastasis with increased CD47 expression, suggesting that CD47 expression was likely acquired during the onset of metastatic dissemination [150].

As previously discussed, inherited genetic variations are likely involved in disrupting the regulation of immune checkpoint molecule expression. Genetic variants in immune checkpoint molecules have been linked to an increased probability of malignancy development, such as breast, bone, cervical, hepatocellular and stomach cancers [12]. The link between inherited genetic variations in immune checkpoint molecules and the likelihood of developing cancer has been thoroughly examined, particularly in the case of *CTLA‐4* and, more broadly, for *PD‐1/PD‐L1*, across various types of cancer. For instance, the presence of the CTLA‐4c.49*A allele has been found to enhance the susceptibility to breast and lung cancers [151, 152], and similarly, the CTLA‐4c.‐1661*G allele in gastric and breast cancers [151, 153].

Thus, genetic variations in CTCs may have the same effect on immune checkpoint molecule production as mutations in cancer genes do in the tumour microenvironment. Since they are shed from the primary tumour and circulate in the bloodstream carrying the genetic information and molecular features of the primary tumour, molecular analysis of CTCs can provide a platform for research into cellular heterogeneities, resistance mechanisms and therapeutic targets in cancer.

Molecular analysis of immune checkpoint molecules in CTCs may provide real‐time information for the clinical management of patients. Though several studies have independently characterised CTCs for driver gene mutations and their mRNA profile, immune‐related gene expression highlights the potential to enhance the understanding of the tumour immune microenvironments and significantly improve treatment decision‐making [102, 131, 151, 154]. Our previous studies have investigated the relationship between genetic alterations in driver cancer genes and their expression profiles, along with immunotherapeutic target molecules, in CTCs from patients with CRC [155, 156]. In these studies, a positive correlation between *KRAS* and *CTLA‐4* gene expression was found. Additionally, it was observed that CTC‐positive patients harbouring *KRAS* mutations exhibited higher *CTLA‐4* gene expression. This suggests that *KRAS* activation may help CTCs evade immune surveillance by modifying *CTLA‐4* expression. These findings provide a preliminary concept for understanding how dysregulation of driver cancer genes may regulate the expression of immune checkpoint molecules, which have a direct role in the initiation and maintenance of cancer gene‐driven tumorigenesis. However, information regarding genetic alterations of driver cancer genes in CTCs and their interaction with the surrounding immune microenvironment, particularly immune checkpoint molecules, remains limited. More detailed studies are required, which can provide new insights into the selection of therapeutic targets at the onset of the disease.

## 9 Concluding Remarks

This review consolidates the state of the current literature on the topic of genetic factors, such as key cancer‐associated genes, including immune checkpoint inhibitors, that drive carcinogenesis, with a focus on CTCs in CRC. This analysis reveals a gap in the literature concerning the genomic profiling of tumour suppressor genes, proto‐oncogenes and immune checkpoint molecules, along with their gene expression analysis in isolated CTCs in CRC. Understanding the interactions between CTCs and these genomic profiles is crucial for uncovering cancer heterogeneity, which could facilitate the identification of personalised therapeutic targets.

## Author Contributions

**Sharmin Aktar:** conceptualization; writing – original draft. **Matthew Masoudi:** writing – original draft. **Dilpreet Moti:** writing – original draft. **Vinod Gopalan:** supervision; writing – review and editing. **Farhadul Islam:** supervision; writing – review and editing. **Alfred King‐yin Lam:** supervision; writing – review and editing.

## Ethics Statement

This study is a literature review. The authors have nothing to report.

## Conflicts of Interest

The authors declare no conflicts of interest.

## Data Availability

The data presented in this study are available in the published paper. “Genetic alterations involved in immune escape mechanisms of circulating tumour cells in colorectal cancer carcinogenesis”.
